# Hepatocellular carcinoma treated with radical resection after endoscopic diagnosis of the extent of bile duct invasion: A case report

**DOI:** 10.1002/deo2.265

**Published:** 2023-07-05

**Authors:** Yudai Sato, Tomoko Tadokoro, Hiroki Yamana, Hiraki Akai, Kei Takuma, Naoki Fujita, Mai Nakahara, Kyoko Oura, Koji Fujita, Joji Tani, Hideki Kamada, Asahiro Morishita, Hideki Kobara, Seiko Kagawa, Reiji Haba, Keiichi Okano, Tsutomu Masaki

**Affiliations:** ^1^ Department of Gastroenterology and Neurology Kagawa University Kagawa Japan; ^2^ Department of Pathology Kagawa University Kagawa Japan; ^3^ Department of Gastroenterological Surgery Kagawa University Kagawa Japan

**Keywords:** bile duct invasion, EUS, hepatocellular carcinoma, peroral cholangioscopy, surgery

## Abstract

Hepatocellular carcinoma invasion of the bile duct is rare and has a poor prognosis. A 77‐year‐old man presented at the emergency department with persistent pain in the right hypochondrium. Blood tests and imaging studies revealed a 70‐mm occupying lesion in the right lobe of the liver and dilated intrahepatic bile ducts. He was diagnosed with obstructive jaundice and cholangitis. Imaging studies showed an internal mass with poor contrast effects. A liver biopsy was performed to confirm the diagnosis and hepatocellular carcinoma was suspected. Endoscopic retrograde cholangiopancreatography, endoscopic ultrasound, and peroral cholangioscopy were performed to determine the treatment strategy. The bile duct invasion did not extend to the porta hepatis; therefore, right hepatic lobectomy and radical resection were performed. Bile duct invasion in hepatocellular carcinoma is rare and often difficult to diagnose by computed tomography or conventional endoscopic retrograde cholangiopancreatography. However, endoscopic ultrasound and peroral cholangioscopy enable safe and accurate diagnosis of the extent of invasion.

## INTRODUCTION

Hepatocellular carcinoma (HCC) presents with various clinical manifestations. However, bile duct invasion and the development of obstructive jaundice are rare.^1^ Many cases are advanced or involve impaired liver function by the time they are detected, and radical surgery is possible only in a limited number of cases. If resection or biliary drainage can be performed for HCC, the prognosis is expected to be equivalent to that of patients without bile duct invasion.^2^

In this report, we describe a case of HCC with bile duct invasion in which radical resection was successfully performed after various imaging studies and peroral cholangioscopy (POCS).

## CASE REPORT

A 77‐year‐old man with a history of hypertension and heavy alcohol consumption presented with epicardial pain and jaundice. The patient had no history of diabetes mellitus. He was afebrile and his vital signs were stable. A physical examination revealed jaundice and moderate epigastric tenderness. His body mass index was calculated to be 20 kg/m^2^.

The following were his laboratory data: total bilirubin: 3.2 mg/dl (fractionated to indirect 2.1 mg/dl, direct 1.1 mg/dl); aspartate aminotransferase: 592 U/L; alanine aminotransferase: 460 U/L; alkaline phosphatase: 332 U/L; γ‐glutamyl transferase: 822 U/L; HBs antigen: negative; HBc antibody: positive; HBs antibody: positive; hepatitis C virus antibody: negative; antimitochondrial antibody: negative; and antinuclear antibody: negative. Tumor markers were α‐fetoprotein: 3754 ng/ml; des‐gamma‐carboxyprothrombin: 11928 mAU/ml; and cancer antigen 19‐9 (CA19‐9): 693.5 U/ml.

**FIGURE 1:**
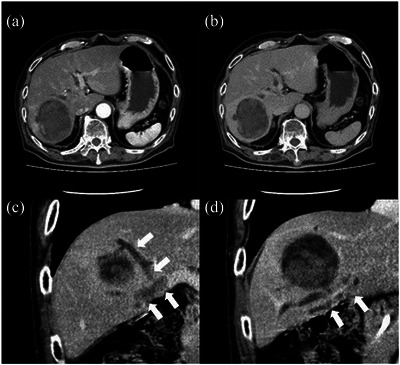
Contrast‐enhanced computed tomography horizontal view (a, b): A 70 × 60 × 55 mm mass is observed in the posterior right lobe of the liver. The limbus had slight early darkening in the arterial phase (a) and lower absorption than the liver parenchyma in the delayed phase (b). Coronal view (c, d): There was a shadow extending from the medial side of the liver tumor, bordering the posterior bile duct, and extending into the hilar bile duct, which had a contrast effect (white arrow).

**FIGURE 2:**
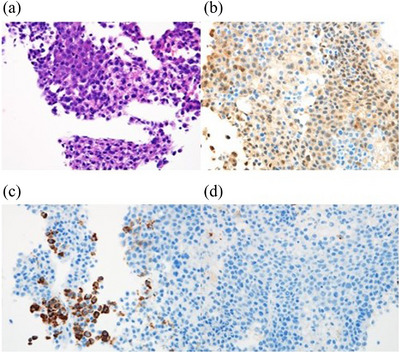
(a) Hematoxylin and eosin staining × 40. Cancer cells with high nuclear density (b–d): Immunohistochemical stain × 40. Positive for arginase‐1 (b) and hepatocytes (c). Negative for cytokeratin‐7 (d) and cytokeratin‐19.

Abdominal ultrasonography revealed a well‐defined, 70 mm hyperechoic mass in the right lobe of the liver and dilatation of the surrounding bile duct. Contrast‐enhanced computed tomography (CT) detected a 70 × 60 × 55 mm mass in the posterior right lobe of the liver, with dilated anterior and posterior bile ducts. The interior of the mass was heterogeneous, and the limbus showed pale early staining in the arterial phase and lower absorption than that of the liver parenchyma in the parallel phase. The tumor was suspected to be medial, bordering the posterior bile duct and extending into the hilar bile duct (Figure 1). Dynamic magnetic resonance imaging showed poor contrast in both phases. Thus, the liver tumor was an atypical imaging finding for HCC; we suspected HCC, mixed HCC invading the bile duct, or HCC complicated by cholangiocarcinoma. A percutaneous liver biopsy was performed for histopathological diagnosis. The liver biopsy specimen showed cancer cells with increased nuclear density. These cells were immunohistochemically positive for arginase‐1 and hepatocytes but negative for cytokeratin 7 and 19. These findings suggested that the main tumor was an HCC (Figure 2).

**FIGURE 3:**
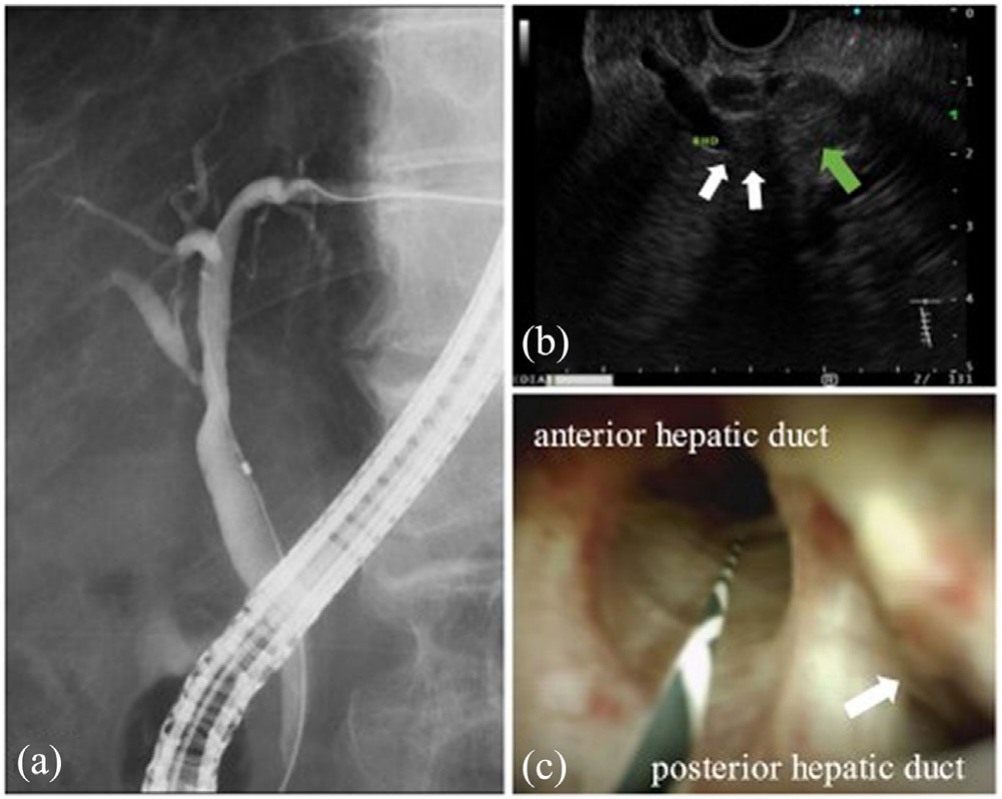
(a) Endoscopic retrograde cholangiopancreatography: stenosis of the right anterior segment bile duct and failure to visualize the posterior segment bile duct. (b) Endoscopic ultrasound: The bile duct was filled with substantial structures (white arrow) and was contiguous with the mass in the posterior segment (green arrow). (c) Peroral cholangioscopy: A villous mass was observed inside the posterior hepatic duct (white arrow); it was difficult to observe from the front. No tumor was observed at the anteroposterior bifurcation of the right hepatic duct to the left‐right hepatic duct bifurcation.

Endoscopic ultrasound revealed a highly echoic structure filling the hepatic duct on observation from the duodenal bulb. The structure was contiguous with a large mass in the posterior segment, which was thought to be bile duct infiltration of the HCC. The extent to which the tumor had invaded the bile duct was unclear (Figure 3b). We performed endoscopic retrograde cholangiopancreatography and POCS to determine the extent of biliary lesion extension and the histopathological diagnosis. A TJF‐Q290V duodenoscope (Olympus, Tokyo, Japan) was then inserted. Endoscopic retrograde cholangiopancreatography showed stenosis of the right anterior segment bile duct and failed to visualize the posterior segment bile duct (Figure 3A). A POCS instrument (SpyGlass DS II, Boston Scientific, Natick, Mass, USA) was inserted into the common bile duct. POCS revealed no abnormalities in the bile duct mucosa from the common bile to the hilar bile duct. The posterior hepatic duct was difficult to observe from the front; however, a villous mass was observed. No tumor was observed in the left hepatic duct, right and left hepatic duct bifurcation, or right hepatic duct anteroposterior bifurcation, and a biopsy confirmed no pathological malignant findings (Figure 3C).

**FIGURE 4:**
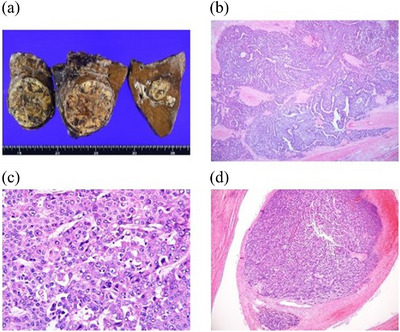
(a) Gross image of the resected right liver lobe. Cancer cells resembling hepatocytes were growing prolifically (b): hematoxylin and eosin staining ×20. Cancer cells with pale cytoplasm were observed (c): Endoscopic ultrasound ×40. The tumor invaded the bile duct (d): Endoscopic ultrasound ×20. The bile duct resection margins were negative.

Positron emission tomography‐CT showed no lymph node metastasis or distant metastasis, and based on POCS findings, the extent of bile duct invasion was judged to be limited to the right hepatic duct. The patient was then referred for right hepatectomy. Histological examination with hematoxylin and eosin staining revealed a moderately to poorly differentiated HCC with a partially pale cytoplasm and a cord‐form structure. The tumor invaded the bile duct; however, the bile duct resection margins were negative, and the tumor could be curatively resected (Figure 4). The patient was provided with adequate informed consent for the publication of this paper.

## DISCUSSION

Liver cancer remains a global health challenge with an increasing incidence worldwide.^3^ HCC is the most common form of liver cancer and accounts for 90% of all liver cancer cases. Despite advances in treatment, the prognosis of HCC remains poor.^4^ Involvement of the portal vein or bile duct is occasionally observed in patients with HCC and has a negative impact on survival.^5^ In particular, HCC invasion of the bile duct is rare, occurring in approximately 0.5%–13% of all cases.^1^ In addition, it has a poorer prognosis than HCC without bile duct invasion.^1^, ^2^ There is no established treatment for bile duct invasion in HCC.^6^ However, there are cases of long‐term survival among patients for whom surgical treatment can be performed.^7^ Overall survival rates vary depending on the initial treatment: rates for surgical resection, transcatheter arterial chemoembolization, systemic chemotherapy, and conservative therapy are 11.5, 6.0, 2.4, and 1.6 months, respectively.^7^ Therefore, the possibility of surgery is likely to play a role in prognosis.

The pathology of HCC with bile duct invasion is inconsistent and can occur at any level of differentiation. Therefore, characteristic findings, such as capsular formation or early enhancement, are often absent on CT or other imaging studies, as in this case.^8^, ^9^ Finally, the tumor was diagnosed as HCC via liver biopsy. To determine a treatment plan, the pathology and extent of bile duct lesions must be evaluated. Conventional biopsy and biliary cytology at the time of the initial endoscopic retrograde cholangiopancreatography did not yield enough specimens to make a diagnosis. Endoscopic ultrasound confirmed that the bile duct lesion was contiguous with the main tumor, whereas POCS confirmed that the bile duct lesion was localized. Based on these findings, radical resection could be performed.

Bile duct invasion in HCC is prone to bleeding, a conventional blind biopsy is risky, and false negatives are common.^10^ Endoscopic ultrasound and POCS are safe and effective methods for evaluating bile duct invasion in HCC. As far as we could find, only a few cases in which POCS was useful in the definitive diagnosis of HCC with bile duct invasion. POCS may be useful not only for primary bile duct tumors but also for determining the operative strategy for HCC with bile duct invasion.

## CONFLICT OF INTEREST STATEMENT

None.
